# Efficacy and Safety of CAR T‐Cell Therapy for Primary and Secondary Central Nervous System Lymphoma: A Systematic Review and Meta‐Analysis

**DOI:** 10.1002/jha2.70407

**Published:** 2026-10-05

**Authors:** Anfal Khan, Aleena Amir Malik, Waqas Kareem, Mohammad Idrees, Kamil Ahmad Kamil, Kinza Irshad, Zainab Mehmood Baig, Anosha Yahya, Wardah Qureshi, Manzoor Ahmad, Bakht Bilanda

**Affiliations:** ^1^ Saidu Medical College Swat Pakistan; ^2^ United Medical and Dental College Karachi Pakistan; ^3^ Rawalpindi Medical University Rawalpindi Pakistan; ^4^ Chief, Department of Internal Medicine Nasrat Bashir Curative Hospital Kandahar Afghanistan; ^5^ Ziauddin University Karachi Pakistan; ^6^ Jinnah Sindh Medical University Karachi Pakistan; ^7^ Growth, Productivity and Entrepreneurship Center of Excellence (GPEC‐X) London UK

**Keywords:** CD‐19 based CART cell therapy, CNS lymphoma, OS, PFS, primary CNS lymphoma, secondary CNS lymphoma, systematic review

## Abstract

**Background:**

Patients with relapsed or refractory primary central nervous system lymphoma (PCNSL) and secondary central nervous system lymphoma (SCNSL) have limited treatment options and poor outcomes. CD19‐directed chimeric antigen receptor (CAR) T‐cell therapy has shown promising activity in this setting; however, the available evidence remains limited and heterogeneous. We performed a systematic review and meta‐analysis to evaluate its efficacy and safety.

**Methods:**

A systematic search of PubMed, Google Scholar, Semantic Scholar, ClinicalTrials.gov and the Cochrane Library was conducted from inception to 31 August 2025. Twenty‐three prospective and retrospective studies involving 547 patients were included. Random‐effects meta‐analyses of proportions were performed to estimate pooled 12‐month overall survival (OS), 12‐month progression‐free survival (PFS), complete response (CR), cytokine release syndrome (CRS) and immune effector cell‐associated neurotoxicity syndrome (ICANS).

**Results:**

The pooled 12‐month OS and PFS rates were 64.5% (95% CI, 49.0%–76.0%) and 51.0% (95% CI, 35.0%–68.0%), respectively. The pooled CR rate was 52% in PCNSL and 40% in SCNSL. CRS occurred in 76% of patients with PCNSL and 77% with SCNSL, while ICANS occurred in 50% and 46%, respectively. Most adverse events were manageable with standard supportive care.

**Conclusions:**

CD19‐directed CAR T‐cell therapy is associated with encouraging survival and response outcomes and an acceptable safety profile in relapsed or refractory PCNSL and SCNSL. However, the available evidence is predominantly retrospective with clinical heterogeneity and relatively short follow‐up, underscoring the need for larger prospective studies with longer follow‐up.

**Trial Registration:**

The authors have confirmed clinical trial registration is not needed for this submission.

## 1 Introduction

Primary central nervous system lymphoma (PCNSL) and secondary central nervous system lymphoma (SCNSL) are aggressive B‐cell malignancies. Current first‐line treatment is based on high‐dose methotrexate (HD‐MTX)‐containing regimens, followed by consolidation with autologous stem‐cell transplantation in eligible patients. Despite these intensive treatment approaches, a substantial proportion of patients eventually develop relapsed or refractory disease. At relapse, therapeutic options include HD‐MTX rechallenge in selected patients, salvage chemoimmunotherapy, and whole‐brain radiotherapy, among others. Nevertheless, outcomes remain poor, with limited survival following disease recurrence or treatment refractoriness [1, 2]. When lymphoma involves the CNS, survival after relapse is often only a matter of months, and there remains an unmet need for more effective therapies [3, 4]. Meanwhile, CD19‐directed CAR T‐cell therapy has dramatically changed outcomes for patients with systemic large B‐cell lymphoma; however, pivotal trials of axicabtagene ciloleucel and tisagenlecleucel largely excluded patients with CNS involvement because of concerns regarding neurotoxicity and uncertain efficacy across the blood–brain barrier [3, 4, 5, 6].

CD19‐directed chimeric antigen receptor (CAR) T‐cell therapy has revolutionized the treatment of relapsed or refractory large B‐cell lymphoma, demonstrating durable responses in heavily pre‐treated patients. However, pivotal clinical trials evaluating axicabtagene ciloleucel, tisagenlecleucel and lisocabtagene maraleucel largely excluded patients with CNS involvement because of concerns regarding neurotoxicity and uncertain efficacy across the blood–brain barrier [3, 4, 5, 6]. Consequently, evidence supporting the use of CAR T‐cell therapy in PCNSL and SCNSL has emerged primarily from retrospective cohorts, registry studies and early prospective experiences [6, 7, 8, 9, 10, 11, 12].

Although these studies have reported encouraging clinical outcomes, the available evidence remains fragmented and is characterized by heterogeneous patient populations, varying CAR T‐cell products, differences in prior treatment exposure and relatively short follow‐up durations [9, 13, 14]. Furthermore, several multicentre cohorts and prospective studies have been published in recent years, substantially expanding the available evidence base. An updated synthesis incorporating these newer studies is therefore warranted to provide a more comprehensive assessment of the efficacy and safety of CD19‐directed CAR T‐cell therapy in patients with CNS lymphoma.

Therefore, we conducted this systematic review and meta‐analysis to comprehensively evaluate the efficacy and safety of CD19‐directed CAR T‐cell therapy in patients with PCNSL and SCNSL. Specifically, we aimed to estimate pooled 12‐month overall survival (OS), 12‐month progression‐free survival (PFS), treatment response and treatment‐related toxicities while summarizing the current evidence to better inform clinical practice and guide future research.

## 2 Methodology

This meta‐analysis was conducted following the Preferred Reporting Items for Systematic Reviews and Meta‐Analyses (PRISMA) guidelines. The study protocol was registered in the International Prospective Register of Systematic Reviews (PROSPERO: ID CRD420251137186). As a study‐level meta‐analysis that synthesized previously published data, it was exempt from Institutional Review Board oversight.

### 2.1 Search Strategy

A systematic search was conducted across multiple electronic databases, including PubMed, Google Scholar, Semantic Scholar, ClinicalTrials.gov and the Cochrane Library, from inception to 31 August 2025, without language restrictions. The search strategy utilized a combination of Medical Subject Headings (MeSH) terms and keywords, including: (‘CAR T‐cell’ OR ‘Chimeric Antigen Receptor T‐cell’) and (‘Central Nervous System Lymphoma’ OR ‘CNS Lymphoma’ OR ‘PCNSL’ OR ‘SCNSL’) AND (‘CD19’). The reference lists of retrieved articles and relevant review papers were manually screened to identify any additional studies not captured by the initial database search.

### 2.2 Study Selection

Titles and abstracts were screened by one investigator, followed by a full‐text review conducted independently by two investigators. Any discrepancies regarding study inclusion were resolved through discussion or by a third reviewer. The inclusion criteria were as follows: (1) Clinical trials (both randomized and non‐randomized) or observational studies (prospective or retrospective), (2) included adult patients with PCNSL or SCNSL, (3) evaluated CD19‐directed CAR T‐cell therapy in patients who had received at least two prior lines of treatment, (4) reported data on at least one of the pre‐specified outcomes: OS, PFS, overall response rate (ORR), complete response (CR) or adverse events including cytokine release syndrome (CRS) and immune effector cell‐associated neurotoxicity syndrome (ICANS).

Although the predefined eligibility criterion specified patients who had received at least two prior lines of therapy, one included study contained a small proportion of patients who had received only one prior line of treatment. This study was retained because the vast majority of participants met the eligibility criteria and separate subgroup data were unavailable.

Exclusion criteria were: (1) Case reports, editorials, reviews or conference abstracts without sufficient outcome data; (2) studies with overlapping patient populations where data could not be disaggregated; (3) studies where the full text was unavailable or did not provide extractable quantitative data for the outcomes of interest. Case series documenting extractable results for two or more patients were eligible for inclusion, while case reports reporting a single patient were excluded due to insufficient data for pooled analysis.

### 2.3 Data Extraction

Two reviewers independently extracted data using a standardized data extraction form. The following information was collected from each included study: Study characteristics (first author, publication year, country of origin and study design); patient characteristics (number of participants, diagnosis [PCNSL or SCNSL] and number of prior lines of therapy); intervention details (type of CD19‐directed CAR T‐cell product); and outcome data, including 12‐month OS, 12‐month PFS, best CR and the incidence of CRS and ICANS. For studies reporting 12‐month OS or PFS directly in the text or tables, these values were extracted directly. When only Kaplan–Meier survival curves were available, 12‐month survival probabilities were extracted using WebPlotDigitizer and independently verified by two reviewers. For studies providing individual patient data or sufficient event and follow‐up information, 12‐month survival estimates were derived using the Kaplan–Meier method where feasible. Studies with insufficient follow‐up to reliably estimate 12‐month survival were not included in the pooled survival analyses. Any discrepancies in data extraction were resolved through discussion and verified by a third reviewer. Corresponding authors were contacted to obtain missing data whenever necessary.

### 2.4 Outcomes

The primary outcomes of this meta‐analysis were: 12‐month OS: The proportion of patients alive at 12 months post‐CAR T‐cell infusion. 12‐month PFS: The proportion of patients alive and free from disease progression at 12 months.

Secondary outcomes included:

*Treatment response*: The proportion of patients achieving a best CR, analysed separately for PCNSL and SCNSL cohorts.

*Safety profile*: The incidence of key adverse events, specifically CRS and ICANS, analysed separately for PCNSL and SCNSL cohorts.

### 2.5 Statistical Analysis

Meta‐analytical computations were performed using R software (R Foundation for Statistical Computing, Vienna, Austria). The pooled measure was the proportion (event rate). Pooled proportions for the primary and secondary outcomes, along with their corresponding 95% confidence intervals (CIs), were calculated using a random‐effects model to account for anticipated clinical and methodological heterogeneity. The Freeman–Tukey double arcsine transformation was applied to stabilize variances for the meta‐analysis of proportions. Heterogeneity among studies was assessed using the *I* ^2^ statistic and interpreted as low (25%–49%), moderate (50%–74%) or high (≥ 75%). A two‐sided *p*‐value < 0.05 was considered statistically significant. Funnel plots were visually inspected to explore potential small‐study effects where feasible. Because several outcomes included a limited number of studies, these assessments were considered exploratory and were interpreted cautiously. Sensitivity analyses were performed using the leave‐one‐out approach to evaluate the robustness of the pooled estimates and determine the influence of individual studies on the overall results.

### 2.6 Risk of Bias Assessment

The methodological quality of the included non‐randomized studies was assessed independently by two reviewers using the *Risk Of Bias In Non‐randomized Studies of Interventions (ROBINS‐I)* tool [15]. The assessment evaluated bias due to confounding, participant selection, intervention classification, deviations from intended interventions, missing data, outcome measurement and selective reporting. Any disagreements were resolved through discussion and, when necessary, consultation with a third reviewer. Overall risk‐of‐bias judgments were assigned according to the ROBINS‐I guidance.

## 3 Results

### 3.1 Search Results

The systematic search identified a total of 1247 records, including 1229 records from electronic databases (PubMed, Cochrane Library, Google Scholar and Semantic Scholar) and 18 records from ClinicalTrials.gov. After removing 355 duplicate records and 10 records removed for other reasons, 882 records remained for title and abstract screening. Of these, 839 records were excluded based on title and abstract, leaving 43 reports for full‐text assessment. All 43 reports were successfully retrieved and assessed for eligibility. Following full‐text review, 20 reports were excluded, including 8 studies with ineligible study designs, 2 reports with overlapping study populations and 10 conference abstracts without sufficient data. Ultimately, [6, 7, 8, 10, 12, 16, 17, 18, 19, 20, 21, 22, 23, 24, 25, 26, 27, 28, 29, 30, 31, 32] studies met the eligibility criteria and were included in both the qualitative systematic review and the quantitative meta‐analysis (Figure 1).

**FIGURE 1:**
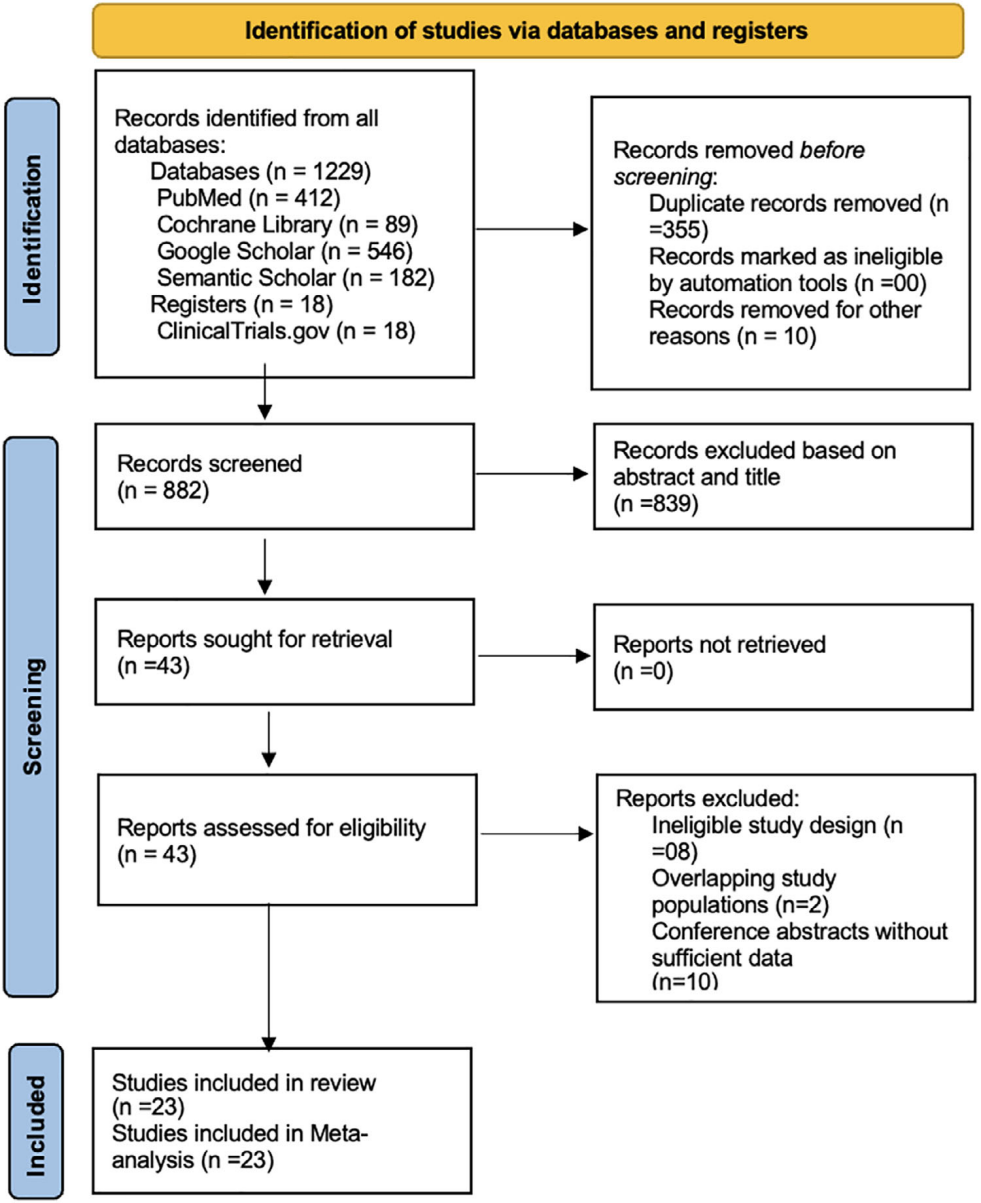
Prisma flow diagram.

### 3.2 Study Characteristics

A total of 23 studies [6, 7, 8, 10, 12, 16, 17, 18, 19, 20, 21, 22, 23, 24, 25, 26, 27, 28, 29, 30, 31, 32] were included in this meta‐analysis, comprising 18 (78%) retrospective and 5 (22%) prospective studies. According to the included studies, a total of 547 patients were evaluated, including patients with PCNSL, SCNSL or both. All included studies evaluated CD19‐directed CAR T‐cell therapy, including axicabtagene ciloleucel, tisagenlecleucel, lisocabtagene maraleucel, CD19‐directed CAR T cells and CD19/CD22‐directed CAR T‐cell therapy. Most patients had received two or more prior lines of therapy, although one study included a small proportion of patients who had received only one prior line of treatment. Where reported, the median follow‐up duration ranged from 5.1 to 37.5 months, with most studies reporting approximately 12 months of follow‐up. The detailed characteristics of the included studies are summarized in Table 1.

**TABLE 1:** Study characteristics.

Author (Year)	Study	Previous lines	Intervention	Sample	Outcomes	Follow‐up time
Abbasi et al. [33]	Retrospective	Three or more line	Axicabtagene ciloleucel (Axi‐cel)	2 SCNSL	Objective response, adverse effects	—
Ahmed et al. [27]	Retrospective	Two, three or four	Axicabtageneciloleucel;	7 SCNSL	PFS, OS, ICANS, CRS	5.1 months (range, 1.6–7.2 months)
Alcantara et al. [24]	Retrospective	Two lines	Axicabtagene ciloleucel (axi‐cel)2, 3 and tisagenlecleucel (tisa‐cel)	9 PCNSL	Median OS, median PFS, objective response, adverse effects (CRS, ICANS)	8.5 months
Alsouqi et al. [32]	Retrospective, multicenter	3 (1–8)	Tisa‐cel, Axi‐Cel, Liso‐cel	113 SCNSL	12 months PFS, OS, CRS, ICANS, Best CR	Median follow‐up 10.7 months
Bennani et al. [29]	Retrospective	Two or three Lines	Axicabtagene Ciloleucel (axi‐cel)	17 SCNSL	RR, EFS, ICANS, CR	median follow‐up of 10.1
Cassanello et al. [22]	Retrospective	3–6 lines	axi‐ cel, tisa‐ cel, liso‐ cel	46 SCNSL and 8 PCNSL	OS, PFS, RR, CRS, ICANS	16.8 months (95% CI: 15.5–29.4)
Choquet et al. [21]	Prospective	Three or more	Axicabtagene ciloleucel (axi‐cel) and lisocabtagene maraleucel (liso‐cel)	27 PCNSL	OS, PFS, RR, CRS, ICANS	19.4 months
Frigault et al. [6]	Retrospective	Three or more lines of therapy	Tisagenlecleucel	8 SCNSL	CR, CRS, ICANS	—
Frigault et al. [31]	Prospective phase 1/2 clinical trial	Previous 2–4 lines	Tisagenlecleucel	12 PCNSL	Response rate, CRS, ICANS	12.2 months (range: 3.64–23.5)
Ghafouri et al. [20]	Prospective	—	Tisagenlecleucel	5 SCNSL	ORR, OS, ICANS	—
Holtzmann et al. [30]	Retrospective	—	Axicabtagene Ciloleucel (axi‐cel)	2 SCNSL	PFS, OR, OS	—
Hui Shi et al. [19]	Retrospective	At least 2 or 3	CART cells	19 PCNSL 8 SCNSL	ORR, PFS, OS, CR	12 months
Abramson et al. [26]	Prospective, multicentre (Phase 1)	Two or three Lines	Lisocabtagene maraleucel (liso‐cel)	7 SCNSL	Median OS, objective response, adverse effects	18.8 months (95% CI: 15.0–19.3)
Wu et al. [18]	Prospective (clinical trial)	Two or three lines	CD19 CAR T cells	4 PCNSL and 9 SCNSL	CRS, ICANS, OR, OS, PFS	14.20 months (range: 1.37–24.17 months)
Wu et al. [17]	Retrospective	> 4 =18, ≤ 4 = 20	CD19 CAR T cells	13 PCNSL and 25 SCNSL	CRS, ICANS, OR, OS, PFS	37.5 months
Karschnia (2022) [8]	Retrospective	Specific distribution not specified	CD19‐directed CAR T‐cell therapy	10 SCNSL	CRS, ICANS, OR, OS, PFS	11.4 months
Karschnia et al. [10]	Retrospective	(Heavily pretreated)	CD19‐directed CAR T‐cell therapy	17 pcnsl 27 scnsl	Response rate, Median OS, PFS: 3,	12.0 ± 5.6 months
Li et al. [25]	Case‐series	Two or more lines	CD19 + CD22	4 SCNSL	OR, CRS, ICANS	—
Nastopil et al. [28]	Retrospective	—	Axicabtagene ciloleucel	21 SCNSL	Median PFS, OR, ICANS, CRS	Median follow‐up of 12.9 months
Saidy et al. [16]	Retrospective	1 line: 6%; 2 lines: 36%; ≥ 3 lines: 58%	Axicabtagene ciloleucel, tisagenlecleucel	100 (84 scnsl, 16 PCNSL	24‐month OS, 24‐month PFS, 24‐month RI, CRS, CRS, ICANS	24.8 months [95% CI: 19–29]
Rui Liu et al. [23]	Retrospective	Two or three lines	CD19 CART cells	6 SCNSL 1 pcnsl	OS (nr), PFS (nr)∖, RR, CRS, ICANS	10.4 months
Siddiqi et al. [7]	Retrospective (Phase 1 clinical trial)	Median: 5 prior lines (range: 2–12)	CD19‐directed CAR T‐cell therapy	5 PCNSL	Complete remission (CR)	—
Zhang et al. [12]	Retrospective	2–3 or 4	Tisagenlecleucel, axicabtagene	15 SCNSL	PFS, OS, CR, ICANS, CR	12 months

### 3.3 Primary Outcome

#### 3.3.1 OS at 12 Months

The pooled analysis demonstrated that the 12‐month OS rate following CD19‐directed CAR T‐cell therapy was 0.645 (95% CI: 0.49–0.76). This indicates that approximately 64% of patients were alive at 12 months after treatment. Moderate heterogeneity was observed among the included studies (*I* ^2^ = 67.6%), suggesting some variability in survival outcomes across studies (Figure 2A). The observed heterogeneity may be attributable to differences in patient characteristics, prior treatments, CAR T‐cell products used and disease subtype (PCNSL vs. SCNSL). Leave‐one‐out analysis could not reduce the heterogeneity by more than 10%.

**FIGURE 2:**
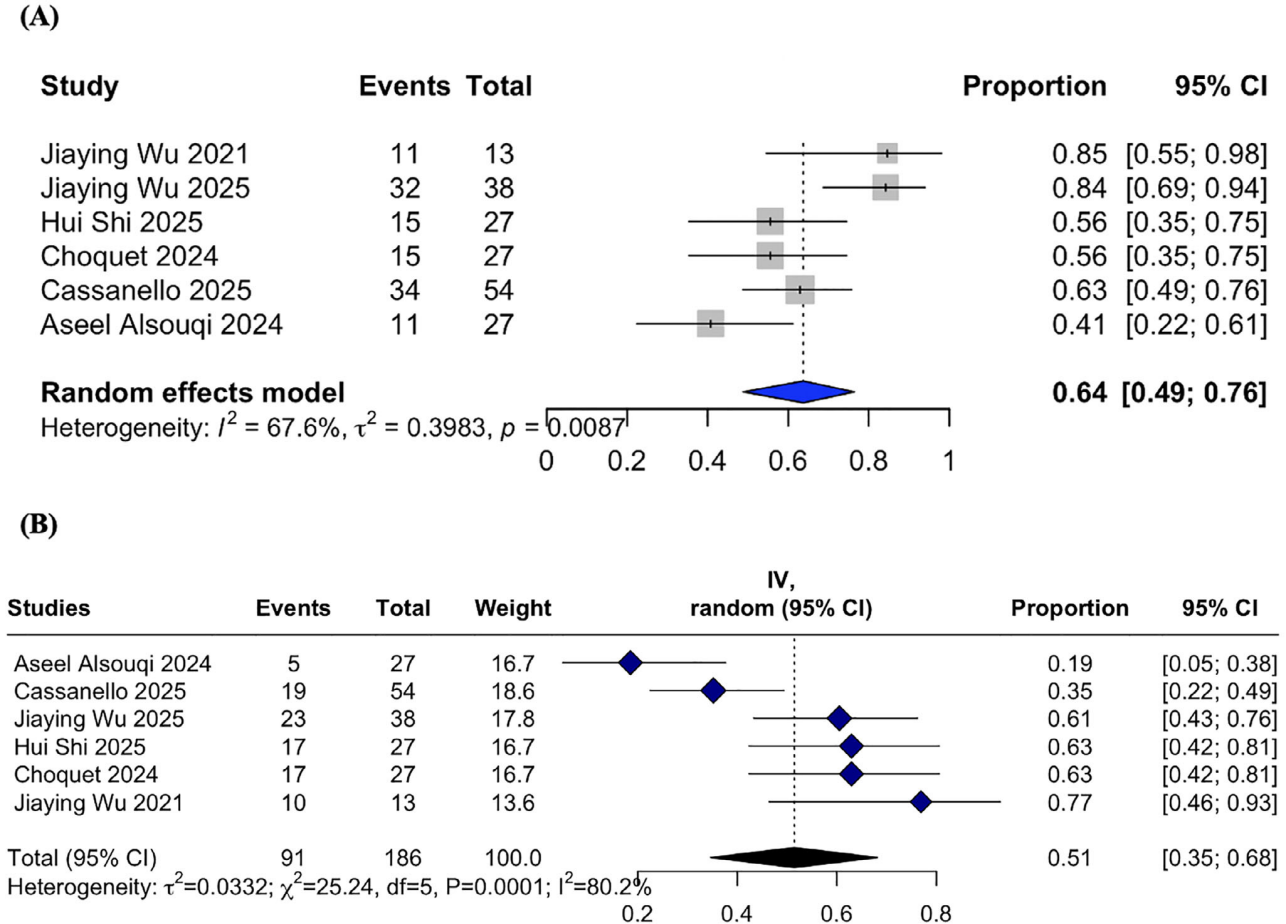
(A) Shows the 12 months overall survival for the CART‐cell therapy group, (B) shows the 12 months progression free survival for the CART‐cell therapy group.

#### 3.3.2 PFS at 12 Months

The pooled 12‐month PFS rate was 0.51 (95% CI: 0.35–0.68). This finding indicates that approximately 51% of patients remained free from disease progression at 1 year following CAR T‐cell therapy. High heterogeneity was detected across studies (*I* ^2^ = 80.2%) (Figure 2B). The observed heterogeneity may be explained by differences in tumour burden, previous treatment regimens and variability in CAR T‐cell constructs used across studies.

### 3.4 Treatment Response

#### 3.4.1 CR in PCNSL

Among patients with PCNSL, the pooled CR rate was **0.52 (95% CI: 0.40–0.64)**. This suggests that approximately **52%** of PCNSL patients achieved complete remission following CAR T‐cell therapy. Low to moderate heterogeneity was observed (*I* ^2^ **= 32%**), indicating relatively consistent results across studies (Figure S1). These findings suggest that CD19‐directed CAR T‐cell therapy can induce encouraging CRs even in heavily pre‐treated patients with PCNSL.

#### 3.4.2 CR in SCNSL

Among patients with secondary CNS lymphoma (SCNSL), the pooled CR rate was 0.40 (95% CI: 0.26–0.55). This indicates that approximately 40% of patients achieved complete remission following CAR T‐cell therapy. Moderate heterogeneity was observed among the included studies (*I* ^2^ = 76%) (Figure S2). The slightly lower CR rate observed in SCNSL compared with PCNSL may reflect the more aggressive disease biology and concomitant systemic disease involvement commonly seen in patients with SCNSL. Leave‐one‐out sensitivity analysis demonstrated that exclusion of the study by Saidy et al. reduced the heterogeneity from 76% to 40%, indicating that this study contributed substantially to the observed between‐study heterogeneity.

### 3.5 Safety Outcomes

#### 3.5.1 Cytokine Release Syndrome

The pooled incidence of CRS among patients with PCNSL was 0.76 (95% CI: 0.59–0.88) (Figure S3). Among patients with SCNSL, the pooled incidence of CRS was 0.77 (95% CI: 0.71–0.82) (Figure S4). No heterogeneity was observed in either the PCNSL or SCNSL analyses (*I* ^2^ = 0% for both). Although CRS was frequently observed, most reported cases were of low to moderate severity and were manageable with standard treatment protocols, including tocilizumab and corticosteroids.

#### 3.5.2 Immune Effector Cell‐Associated Neurotoxicity Syndrome

The pooled incidence of ICANS was 0.50 (95% CI: 0.37–0.62) among patients with PCNSL and 0.46 (95% CI: 0.35–0.57) among patients with SCNSL (Figures S5 and S6). No heterogeneity was observed among studies contributing to the PCNSL analysis (*I* ^2^ = 0%), whereas moderate heterogeneity was observed in the SCNSL analysis (*I* ^2^ = 50.0%). Despite the relatively high incidence of ICANS, most events were reported to be reversible and manageable with appropriate supportive care.

### 3.6 Publication Bias

Visual inspection of the funnel plots for the primary and secondary outcomes (12‐month OS, 12‐month PFS, CR, CRS and ICANS) did not demonstrate obvious asymmetry (Figures S7–S10). However, because several outcomes included a limited number of studies, the interpretability of funnel plots is limited, and publication bias cannot be excluded. Therefore, these findings should be interpreted with caution.

### 3.7 Risk of Bias Assessment

Risk of bias was assessed for all 23 included studies using the ROBINS‐I tool. Overall, seven studies were judged to have a low risk of bias, seven had a moderate risk of bias and nine had a serious risk of bias. The most frequent concerns were related to confounding, selection of participants, deviations from intended interventions and measurement of outcomes. Several studies were considered to have serious overall risk of bias because of their retrospective design, potential confounding and limitations in participant selection and outcome assessment. The risk‐of‐bias assessments for each study and individual ROBINS‐I domain are presented in Table S1.

## 4 Discussion

This meta‐analysis evaluated the efficacy and safety of CD19‐directed CAR T‐cell therapy in patients with PCNSL and SCNSL. The pooled results from 23 studies involving 547 patients suggested encouraging survival outcomes, favourable response rates and a manageable toxicity profile in this heavily pre‐treated population.

### 4.1 Survival Outcomes

The pooled analysis demonstrated a 12‐month OS rate of 64.5% and a 12‐month PFS rate of 51%, suggesting that CD19‐directed CAR T‐cell therapy is associated with encouraging survival outcomes in patients with relapsed or refractory CNS lymphoma. These findings are comparable with clinical trials of CAR T‐cell products, including axicabtagene ciloleucel, tisagenlecleucel and lisocabtagene maraleucel, which have reported durable responses in relapsed or refractory large B‐cell lymphoma [26, 34, 35], and are consistent with the meta‐analysis by Cook et al., which demonstrated favourable response rates and a manageable safety profile in patients with PCNSL and SCNSL treated with CAR T‐cell therapy [9].

Despite these encouraging findings, disease progression remains common in a subset of patients. Possible explanations include the aggressive biological behaviour of CNS lymphoma, limited persistence of CAR T cells in some patients and immune evasion mechanisms within the tumour microenvironment.

### 4.2 Treatment Response

In this meta‐analysis, the pooled CR rate was 52% for PCNSL and 40% for SCNSL, suggesting encouraging antitumor activity of CD19‐directed CAR T‐cell therapy in patients with relapsed or refractory CNS lymphoma. These response rates are particularly notable given that most patients included in the analyzed studies had previously received multiple lines of therapy.

Similar response rates have been reported in previous studies evaluating CAR T‐cell therapy in aggressive B‐cell lymphomas. For example, the pivotal ZUMA‐1 trial demonstrated high response rates with axicabtagene ciloleucel in patients with refractory large B‐cell lymphoma [32]. Likewise, the TRANSCEND NHL 001 trial reported durable responses with lisocabtagene maraleucel in heavily pretreated patients [27]. Additionally, Siddiqi et al. reported that CD19‐directed CAR T‐cell therapy could produce rapid and durable responses in patients with relapsed or refractory B‐cell lymphomas [36]. Similarly, Frigault et al. demonstrated that CAR T‐cell therapy can induce meaningful responses in patients with secondary CNS lymphoma, supporting the ability of CAR T cells to target malignant B cells within the central nervous system [6].

The slightly lower CR rate observed in SCNSL compared with PCNSL may reflect differences in disease biology. Secondary CNS lymphoma frequently occurs in the setting of systemic lymphoma and may be associated with a higher tumor burden and more aggressive disease characteristics, which could adversely affect treatment response.

### 4.3 Safety Outcomes

The safety analysis demonstrated that CRS occurred in approximately 76%–77% of patients, while ICANS occurred in 46%–50% of patients. Although these adverse events were relatively common, most were reported to be manageable with established supportive care strategies, including tocilizumab and corticosteroids where appropriate.

Neurotoxicity has historically been a major concern when administering CAR T‐cell therapy to patients with CNS involvement. In the present meta‐analysis, the incidences of CRS and ICANS were consistent with those reported in previous studies evaluating CAR T‐cell therapy in CNS lymphoma. Frigault et al. similarly reported that patients with secondary CNS lymphoma treated with tisagenlecleucel did not experience unexpectedly high rates of neurotoxicity and that treatment was generally feasible with appropriate monitoring and supportive care [6]. Although these findings are broadly consistent with reports in systemic lymphoma, direct comparisons should be interpreted with caution because our meta‐analysis did not include a non‐CNS comparator cohort. Overall, the available evidence suggests that CAR T‐cell therapy can be safely administered to patients with CNS lymphoma when appropriate monitoring and management strategies are implemented.

### 4.4 Strengths

This study has several strengths. To our knowledge, it represents one of the most comprehensive and up‐to‐date systematic reviews and meta‐analyses evaluating CD19‐directed CAR T‐cell therapy in patients with PCNSL and SCNSL. By incorporating recently published prospective studies and multicentre cohorts, this meta‐analysis provides a more comprehensive synthesis of the available evidence than previous reviews. Furthermore, separate pooled analyses were performed for treatment response and safety outcomes in PCNSL and SCNSL, allowing a more clinically meaningful assessment of disease‐specific outcomes. The methodological rigor of this study was strengthened by adherence to PRISMA guidelines, prospective protocol registration, duplicate data extraction and formal risk‐of‐bias assessment using the ROBINS‐I tool [33].

### 4.5 Heterogeneity and Limitations

Moderate heterogeneity was observed across several pooled outcomes in this meta‐analysis. This variability may reflect differences in study design, patient characteristics, disease subtype (PCNSL vs. SCNSL), CAR T‐cell constructs and treatment protocols among the included studies. Many studies were retrospective or involved small sample sizes, which may introduce selection bias and reduce the overall certainty of the available evidence.

Additional limitations include variability in prior treatment exposure and differences in follow‐up duration across studies. Furthermore, multiple CAR T‐cell products were used across studies, including axicabtagene ciloleucel, tisagenlecleucel and lisocabtagene maraleucel, which may influence both efficacy and toxicity outcomes [6, 26, 34, 36]. Separate pooled analyses for 12‐month OS and PFS in PCNSL and SCNSL were not feasible because most included studies reported combined survival outcomes without sufficient disease‐specific subgroup data, limiting disease‐specific meta‐analysis.

Notably, the median follow‐up across the included studies was relatively short (approximately 12 months), which limits the ability to draw strong conclusions about long‐term durability of response, late relapse and long‐term safety. **In addition, although publication bias was assessed using funnel plots, the limited number of studies contributing to several outcomes reduced the interpretability of these analyses. Therefore, the findings should be interpreted with appropriate caution. Future well‐designed prospective multicentre studies with larger patient populations, standardized outcome reporting, and longer follow‐up are needed to better define the long‐term efficacy and safety of CD19‐directed CAR T‐cell therapy in patients with CNS lymphoma**.

## 5 Conclusion

Overall, the available evidence suggests that CD19‐directed CAR T‐cell therapy is a feasible and promising therapeutic option for patients with relapsed or refractory PCNSL and SCNSL. The therapy is associated with encouraging survival outcomes, favourable CR rates and a manageable safety profile. However, these findings should be interpreted with caution because the available evidence is predominantly derived from retrospective studies with clinical heterogeneity and relatively short follow‐up. Further prospective clinical trials with larger patient populations and longer follow‐up are required to better define the role of CD19‐directed CAR T‐cell therapy in the treatment of CNS lymphoma.

## Author Contributions

**Anfal Khan**: conceptualization, data curation, writing – original draft. **Aleena Amir Malik**: supervision, project administration, data curation, writing – review and editing, visualization. **Waqas Kareem**: formal analysis, data curation, project administration, writing – review and editing. **Mohammad Idrees**: methodology, data extraction, validation. **Kamil Ahmad Kamil**: methodology, writing – review and editing. **Kinza Irshad**: data curation, formal analysis. **Zainab Mehmood Baig**: investigation, literature search. **Anosha Yahya**: data curation, project administration, writing – review and editing. **Wardah Qureshi**: critical review, editing. **Manzoor Ahmad**: statistical analysis, validation. **Bakht Bilanda**: data extraction, proofreading.

## Funding

The authors have nothing to report.

## Ethics Statement

This study is a systematic review and meta‐analysis of previously published studies and does not involve human participants or animals directly. Therefore, ethical approval and informed consent were not required.

## Conflicts of Interest

The authors declare no conflicts of interest.

## Supporting information




**Table S1**: Shows the RoBins‐1 assessment for all the included studies.
**Figure S1**: Shows the number of participants with complete response for PCNSL.
**Figure S2**: Shows the number of participants with complete response for SCNSL.
**Figure S3**: Shows the number of participants having CRS (Cytokines release syndrome) in PCNSL.
**Figure S4**: Shows the number of participants having CRS (Cytokines release syndrome) in SCNSL.
**Figure S5**: Shows the number of participants with ICANS (Immune Effector Cell‐Associated Neurotoxicity Syndrome in PCNSL patients.
**Figure S6**: Shows the number of participants with ICANS (Immune Effector Cell‐Associated Neurotoxicity Syndrome in SCNSL patients.M.
**Figure S7**: Funnel Plot OS and PFS.
**Figure S8**: Funnel plot best CR.
**Figure S9**: Funnel Plots CRS.
**Figure S10**: Funnel Plots ICANS.

## Data Availability

All data analysed in this study are derived from previously published articles and are included within the manuscript. Further details are available from the corresponding author upon reasonable request.

## References

[1] A. J. M. Ferreri , G. Illerhaus , J. K. Doorduijn , et al., “Primary Central Nervous System Lymphomas: EHA‐ESMO Clinical Practice Guideline for Diagnosis, Treatment and Follow‐Up,” Annals of Oncology 35 (2024): 491–507, 10.1016/j.annonc.2023.11.010. 38839484

[2] K. Cwynarski , T. Cummin , W. Osborne , et al., “Management of Secondary Central Nervous System Lymphoma,” British Journal of Haematology 200, no. 2 (2023): 160–169, 10.1111/bjh.18539. 36408800

[3] P. Karschnia , J. Blobner , N. Teske , et al., “CAR T‐Cells for CNS Lymphoma: Driving Into New Terrain?,” Cancers 13, no. 10 (2021): 2503, 10.3390/cancers13102503. PMC8161128 34065471

[4] X. Wu , X. Zhang , R. Xun , M. Liu , Z. Sun , and J. Huang , “Efficacy and Safety of Axicabtagene Ciloleucel and Tisagenlecleucel Administration in Lymphoma Patients With Secondary CNS Involvement: A Systematic Review,” Frontiers in Immunology 12 (2021): 693200, 10.3389/fimmu.2021.693200. PMC8287648 34290712

[5] N. Asghar , A. Masood , A. Dhaliwal , et al., “Chimeric Antigen Receptor T‐Cell (CAR T‐Cell) Therapy for Primary and Secondary Central Nervous System Lymphoma: A Systematic Review of Literature,” Clinical Lymphoma Myeloma and Leukemia 23, no. 1 (2023): 15–21, 10.1016/j.clml.2022.09.008. 36328891

[6] M. J. Frigault , J. Dietrich , M. Martinez‐Lage , et al., “Tisagenlecleucel CAR T‐Cell Therapy in Secondary CNS Lymphoma,” Blood 134, no. 11 (2019): 860–866, 10.1182/blood.2019001694. PMC7022436 31320380

[7] T. Siddiqi , X. Wang , M. S. Blanchard , et al., “CD19‐Directed CAR T‐Cell Therapy for Treatment of Primary CNS Lymphoma,” Blood Advances 5, no. 20 (2021): 4059–4063, 10.1182/bloodadvances.2020004106. PMC8945630 34492703

[8] P. Karschnia , K. Rejeski , M. Winkelmann , et al., “Toxicities and Response Rates of Secondary CNS Lymphoma After Adoptive Immunotherapy With CD19‐Directed Chimeric Antigen Receptor T Cells,” Neurology 98, no. 21 (2022): 884–889, 10.1212/WNL.0000000000200608. PMC9169944 35351785

[9] M. R. Cook , C. S. Dorris , K. H. Makambi , et al., “Toxicity and Efficacy of CAR T‐Cell Therapy in Primary and Secondary CNS Lymphoma: A Meta‐Analysis of 128 Patients,” Blood Advances 7, no. 1 (2023): 32–39, 10.1182/bloodadvances.2022008525. PMC9813524 36260735

[10] P. Karschnia , I. C. Arrillaga‐Romany , A. Eichler , et al., “Neurotoxicity and Management of Primary and Secondary Central Nervous System Lymphoma After Adoptive Immunotherapy With CD19‐Directed Chimeric Antigen Receptor T‐Cells,” Neuro‐Oncology 25, no. 12 (2023): 2239–2249, 10.1093/neuonc/noad118. PMC10708936 37402650

[11] N. Epperla , L. Feng , N. N. Shah , et al., “Outcomes of Patients With Secondary Central Nervous System Lymphoma Following CAR T‐Cell Therapy: A Multicenter Cohort Study,” Journal of Hematology & Oncology 16, no. 1 (2023): 111, 10.1186/s13045-023-01508-3. PMC10633964 37946255

[12] H. Zhang , Z. Yan , Y. Wang , et al., “Efficacy and Safety of CD19‐Specific CAR‐T Cell‐Based Therapy in Secondary Central Nervous System Lymphoma,” Frontiers in Immunology 13 (2022): 965224, 10.3389/fimmu.2022.965224. PMC9437350 36059496

[13] J. Zhou , Z. Wang , H. Wang , Y. Cao , and G. Wang , “Sustained Efficacy of Chimeric Antigen Receptor T‐Cell Therapy in Central Nervous System Lymphoma: A Systematic Review and Meta‐Analysis of Individual Data,” Frontiers in Pharmacology 14 (2024): 1331844, 10.3389/fphar.2023.1331844. PMC10847290 38328579

[14] L. Lv , Y. Wu , H. Shi , et al., “Efficacy and Safety of Chimeric Antigen Receptor T‐Cells Treatment in Central Nervous System Lymphoma: A PRISMA‐Compliant Single‐Arm Meta‐Analysis,” Cancer Immunology, Immunotherapy 72, no. 1 (2023): 211–221, 10.1007/s00262-022-03246-w. PMC10991213 35796863

[15] J. A. Sterne , M. A. Hernán , B. C. Reeves , et al., “ROBINS‐I: A Tool for Assessing Risk of Bias in Non‐Randomised Studies of Interventions,” BMJ 355 (2016): i4919, 10.1136/bmj.i4919. PMC5062054 27733354

[16] A. O. Saidy , C. Peczynski , and C. Thieblemont , “Efficacy and Safety of CAR T‐Cell Therapy in Patients With Primary or Secondary CNS Lymphoma: A Study on Behalf of the EBMT and the GoCART Coalition,” HemaSphere 9, no. 5 (2025): e70146, 10.1002/hem3.70146. PMC12093105 40400509

[17] J. Wu , W. Liu , Y. Cao , et al., “Clinical Outcomes of Chimeric Antigen Receptor T‐Cell Therapy Following Autologous Hematopoietic Stem Cell Transplantation in 38 Patients With Refractory/Relapsed Primary or Secondary Central Nervous System Lymphoma,” Cancer Immunology, Immunotherapy 74, no. 1 (2024): 17, 10.1007/s00262-024-03855-7. PMC11554992 39527142

[18] J. Wu , F. Meng , Y. Cao , et al., “Sequential CD19/22 CAR T‐cell Immunotherapy Following Autologous Stem Cell Transplantation for Central Nervous System Lymphoma,” Blood Cancer Journal 11, no. 7 (2021): 131, 10.1038/s41408-021-00523-2. PMC8282870 34267187

[19] H. Shi , P. Zheng , Z. Fu , et al., “Whole Brain Radiotherapy Combined With CART‐Cell Therapy for Relapsed/Refractory Central Nervous System B‐Cell Lymphoma,” Annals of Hematology 104, no. 4 (2025): 2495–2505, 10.1007/s00277-025-06378-y. PMC12052746 40278918

[20] S. Ghafouri , J. Timmerman , S. Larson , and M. D. Mead , “Axicabtagene Ciloleucel CAR T‐Cell Therapy for Relapsed/Refractory Secondary CNS Non‐Hodgkin Lymphoma: Comparable Outcomes and Toxicities, But Shorter Remissions May Warrant Alternative Consolidative Strategies?,” Bone Marrow Transplantation 56, no. 4 (2021): 974–977, 10.1038/s41409-020-01099-4. PMC8035067 33168933

[21] S. Choquet , C. Soussain , N. Azar , et al., “CAR T‐Cell Therapy Induces a High Rate of Prolonged Remission in Relapsed Primary CNS Lymphoma: Real‐Life Results of the LOC Network,” American Journal of Hematology 99, no. 7 (2024): 1240–1249, 10.1002/ajh.27316. 38586986

[22] G. Cassanello , E. Luttwak , S. Brown , et al., “Outcomes of CD19 CAR ‐T Therapy in Central Nervous System Lymphoma: Insights From a Multicentre Experience,” British Journal of Haematology 207, no. 2 (2025): 525–534, 10.1111/bjh.20234. PMC13482356 40589238

[23] R. Liu , Q. Cheng , L. Kang , et al., “CD19 or CD20 CAR T Cell Therapy Demonstrates Durable Antitumor Efficacy in Patients With Central Nervous System Lymphoma,” Human Gene Therapy 33, no. 5–6 (2022): 318–329, 10.1089/hum.2021.249. 35152726

[24] M. Alcantara , C. Houillier , M. Blonski , et al., “CAR T‐Cell Therapy in Primary Central Nervous System Lymphoma: The Clinical Experience of the French LOC Network,” Blood 139, no. 5 (2022): 792–796, 10.1182/blood.2021012932. PMC8814680 34871363

[25] T. Li , L. Zhao , Y. Zhang , et al., “CAR T‐Cell Therapy Is Effective But Not Long‐Lasting in B‐Cell Lymphoma of the Brain,” Frontiers in Oncology 10 (2020): 1306, 10.3389/fonc.2020.01306. PMC7438944 32903866

[26] J. S. Abramson , M. L. Palomba , L. I. Gordon , et al., “Lisocabtagene Maraleucel for Patients With Relapsed or Refractory Large B‐Cell Lymphomas (TRANSCEND NHL 001): A Multicentre Seamless Design Study,” Lancet 396, no. 10254 (2020): 839–852, 10.1016/S0140-6736(20)31366-0. 32888407

[27] G. Ahmed , M. Hamadani , and N. N. Shah , “CAR T‐Cell Therapy for Secondary CNS DLBCL,” Blood Advances 5, no. 24 (2021): 5626–5630, 10.1182/bloodadvances.2021005292. PMC8714710 34551065

[28] L. J. Nastoupil , M. D. Jain , L. Feng , et al., “Standard‐of‐Care Axicabtagene Ciloleucel for Relapsed or Refractory Large B‐Cell Lymphoma: Results From the US Lymphoma CAR T Consortium,” Journal of Clinical Oncology 38, no. 27 (2020): 3119–3128, 10.1200/JCO.19.02104. PMC7499611 32401634

[29] N. N. Bennani , M. J. Maurer , L. J. Nastoupil , et al., “Experience With Axicabtagene Ciloleucel (Axi‐Cel) in Patients With Secondary CNS Involvement: Results From the US Lymphoma CAR T Consortium,” Blood 134, no. S1 (2019): 763–763, 10.1182/blood-2019-129097.

[30] N. G. Holtzman , H. Xie , S. Bentzen , et al., “Immune Effector Cell–Associated Neurotoxicity Syndrome After Chimeric Antigen Receptor T‐Cell Therapy for Lymphoma: Predictive Biomarkers and Clinical Outcomes,” Neuro‐Oncology 23, no. 1 (2021): 112–121, 10.1093/neuonc/noaa183. PMC7850044 32750704

[31] M. J. Frigault , J. Dietrich , K. Gallagher , et al., “Safety and Efficacy of Tisagenlecleucel in Primary CNS Lymphoma: A Phase 1/2 Clinical Trial,” Blood 139, no. 15 (2022): 2306–2315, 10.1182/blood.2021014738. PMC9012129 35167655

[32] A. Alsouqi , G. Ahmed , J. Wang , et al., “Chimeric Antigen Receptor T‐Cell Therapy in Secondary Central Nervous System Lymphoma: A Multicenter Analysis,” American Journal of Hematology 99, no. 8 (2024): 1624–1627, 10.1002/ajh.27354. 38769663

[33] A. Abbasi , S. Peeke , N. Shah , et al., “Axicabtagene Ciloleucel CD19 CAR‐T Cell Therapy Results in High Rates of Systemic and Neurologic Remissions in Ten Patients With Refractory Large B Cell Lymphoma Including Two With HIV and Viral hepatitis,” Journal of Hematology & Oncology 13, no. 1 (2020), 10.1186/s13045-019-0838-y. PMC6942268 31900191

[34] S. S. Neelapu , F. L. Locke , N. L. Bartlett , et al., “Axicabtagene Ciloleucel CAR T‐Cell Therapy in Refractory Large B‐Cell Lymphoma,” New England Journal of Medicine 377, no. 26 (2017): 2531–2544, 10.1056/NEJMoa1707447. PMC5882485 29226797

[35] S. J. Schuster , M. R. Bishop , C. S. Tam , et al., “Tisagenlecleucel in Adult Relapsed or Refractory Diffuse Large B‐Cell Lymphoma,” New England Journal of Medicine 380, no. 1 (2019): 45–56, 10.1056/NEJMoa1804980. 30501490

[36] F. L. Locke , A. Ghobadi , C. A. Jacobson , et al., “Long‐Term Safety and Activity of Axicabtagene Ciloleucel in Refractory Large B‐Cell Lymphoma (ZUMA‐1): A Single‐Arm, Multicentre, Phase 1–2 Trial,” Lancet Oncology 20, no. 1 (2019): 31–42, 10.1016/S1470-2045(18)30864-7. PMC6733402 30518502

[37] G. Losi , A. Mussetti , M. Peña , P. Lopez‐Pereira , A. Sureda , and S. Novelli , “Anti‐CD19 CAR T‐Cell Therapy for Primary and Secondary CNS Lymphomas,” Bone Marrow Transplantation 60, no. 3 (2025): 259–269, 10.1038/s41409-024-02496-9. 39674850

[38] P. Karschnia , J. T. Jordan , D. A. Forst , et al., “Clinical Presentation, Management, and Biomarkers of Neurotoxicity After Adoptive Immunotherapy With CAR T Cells,” Blood 133, no. 20 (2019): 2212–2221, 10.1182/blood-2018-12-893396. 30808634

