# Supplementary material for: Efficacy and Safety of CAR T‐Cell Therapy for Primary and Secondary Central Nervous System Lymphoma: A Systematic Review and Meta‐Analysis
Source: EJHaem. 2026 Oct 5;7(5):e70407. doi: 10.1002/jha2.70407 (PMC13639112; doi:10.1002/jha2.70407)
Supplement: Supplementary file 1 [file JHA2-7-e70407-s001.docx]

Supplementary File

**Supplementary Table 1**: Shows the RoBins-1 assessment for all the included studies.

| **Study (First Author, Year)** | **Confounding** | **Selection of Participants** | **Classification of Interventions** | **Deviations from Interventions** | **Missing Data** | **Measurement of Outcomes** | **Selection of Reported Result** | **Overall RoB** |
| --- | --- | --- | --- | --- | --- | --- | --- | --- |
| Abbasi et al. 2020 (24) | Moderate | Low | Moderate | Moderate | Moderate | Moderate | Low | Moderate |
| Ahmed et al., 2021 (26) | Serious | Moderate/Serious | Low/Moderate | Moderate | Moderate | Low/Moderate | Moderate | Serious |
| Alcantara et al. 2022(22) | Moderate | Moderate | Low | Low | Low | Low | Low | Low |
| Alsouqi et al., 2024 (32) | Low | Low | Low | Low | Moderate | Low | Low | Low |
| Bennani et al. 2019(28) | Serious | Serious | Low/Moderate | Low | Moderate | Low/ Moderate | Moderate | Serious |
| Cassanello 2025(20) | Moderate | Moderate | Moderate | Moderate | Low/Moderate | Low | Moderate | Moderate |
| Choquet 2024(19) | Moderate | Moderate | Low | Low/Mod | Low/Mod | Moderate | Low/Mod | Moderate |
| Frigault 2019(4) | Serious | Serious | Low | Moderate | Moderate | Moderate | Serious | Serious |
| Frigault 2022(30) | Serious | Moderate | Low | Moderate | Low | Low/Mod | Low/Mod | Serious |
| Ghafouri et al. 2021 (18) | Serious | Moderate/Serious | Low/Moderate | Moderate | Moderate | Low | Moderate | Serious |
| Holtzmann et al. 2021(29) | Moderate | Moderate | Low | Moderate | Low | Low | Moderate | Moderate |
| Hui Shi 2025(17) | Serious | Moderate/Serious | Moderate | Moderate | Low/Moderate | Low | Low/Moderate | Moderate |
| Jeremy S Abramson et al. 2020 (25) | Moderate | Low/Mod | Low | Low/Mod | Low | Low/Mod | Low | Low |
| Jiaying Wu 2021(16) | Moderate | Moderate | Low | Low | Low | Low | Low/Moderate | Low |
| Jiaying Wu 2025 (15) | Moderate/Serious | Moderate | Low/Moderate | Moderate | Low/Moderate | Low | Low/Moderate | Moderate |
| Karschnia 2022 (6) | Serious | Moderate | Low/Moderate | Low | Low/Moderate | Low | Low | Serious |
| Karschnia 2023(8) | Moderate | Moderate | Low | Low | Low | Low | Low | Low |
| **Li et al 2020**  (23) | Serious | Moderate | Low/Moderate | Moderate | Low | Low | Low/Moderate | Serious |
| Nastopil et al. 2020 (27) | Moderate–Serious | Moderate | Low/Moderate | Low | Low/Moderate | Low | Low | Moderate |
| Ossami Saidy 2025(13) | Moderate | Low/Moderate | Low/Moderate | Low | Low | Moderate | Low | Low |
| Rui Liu 2022(21) | Moderate | Low | Low | Low | Low/Moderate | Low | Low | Low |
| Siddiqi 2021(5) | Serious | Serious | Low/Moderate | Low/Moderate | Moderate | Low/Moderate | Moderate | Serious |
| Zhang et al., 2022 (10) | Serious | Moderate/Serious | Moderate | Moderate | Moderate | Low/Moderate | Moderate | Serious |

**Supplementary Figure 1:** Shows the number of participants with complete response for PCNSL

**Supplementary Figure 2:** Shows the number of participants with complete response for SCNSL

**CRS PCNSL**

**Supplementary Figure 3:** Shows the number of participants having CRS (Cytokines release syndrome) in PCNSL patients

**CRS SCNS**

**Supplementary Figure 4:** Shows the number of participants having CRS (Cytokines release syndrome) in SCNSL patients

 **Supplementary Figure 5: Shows the number of participants with ICANS (Immune Effector Cell-Associated Neurotoxicity Syndrome in PCNSL patients.**

**ICAN SCNSL**

## **Supplementary Figure 6: Shows the number of participants with ICANS (Immune Effector Cell-Associated Neurotoxicity Syndrome in SCNSL patients.**

**Funnel Plot OS and PFS:**

**Supplementary Figure 7 Funnel Plot OS and PFS**

**Supplementary Figure 8 Funnel plot best CR**

**Supplementary Figure 9 Funnel Plots CRS**

**Supplementary Figure 10 Funnel Plots ICANS**
